# Analysis of Iron Metabolism in Chronic Chagasic Cardiomyopathy

**DOI:** 10.5935/abc.20190006

**Published:** 2019-02

**Authors:** Carla Paixão Miranda, Fernando Antônio Botoni, Maria do Carmo Pereira Nunes, Manoel Otávio da Costa Rocha

**Affiliations:** 1 Universidade Federal de Minas Gerais, Belo Horizonte, MG - Brazil; 2 Hospital das Clínicas - Universidade Federal de Minas Gerais, Belo Horizonte, MG - Brazil

**Keywords:** Iron Metabolism Disorders, Chagas Disease, Chagas Cardiomyopathy, Inflammation, Anemia

## Abstract

Changes in iron metabolism in heart failure (HF) have been described as an important prognostic marker.

To check if the markers of iron kinetics are related to the morbidity and etiology of chagasic cardiomyopathy.

Patients with Chronic Chagasic Cardiomyopathy (CCC, n = 40), with indeterminate form (IND, n = 40), besides non-chagasic cardiomyopathy (NCh, n = 40).

The mean age was 50.98 ± 5.88 in CCC, 50% were male, 49.68 ± 5.28 in IND, 52.2% were male, and 49.20 ± 10.09 in NCh, 12.5% were male. Lower levels of iron (FeSe) were observed in the CCC groups (93.15 ± 36.53), when compared to IND (125.30 ± 22.79) and NCh (114.77 ± 18.90) (p = 0.0004), lower IST transferrin saturation index in CCC (29.48 ± 6.59), when compared to IND (30.95 ± 7.06) and in the NCh group (39.70 ± 7.54) p = 0.0001), total binding capacity of the lower CTLF iron in the CCC group (297.30 ± 36.46), when compared to the IND group (196.52 ± 56.95) and the NCh group (275.18 ± 33, 48) (p = 0.0001), lower ferritin in the CCC group (134.55, 1.56-42.36), when compared to the IND group (156,25, 1,72-42,20) and the NCh group (112.95, 2.88-42.66) (p = 0.0004). It was also observed that FeSe (95% CI 1.00-1.04, p = 0.0014), IST (95% CI 1.02-1.22) (p = 0.0012) and gender (95% CI 1.07-14.43 p = 0.0038) were independently associated with the degree of ventricular dysfunction in chagasic cardiomyopathy.

CCC patients showed greater change in iron metabolism regarding the indeterminate form and other forms of cariomyopathies.

## Introduction

Functional iron deficiency (Fe) can be defined as the imbalance between the required amount of Fe for hemoglobin synthesis and its supply.^1^ It occurs in the absence of Fe stock, a characteristic of iron deficiency anemia (FA), and in the presence of blockade of Fe homeostasis, as in anemia of inflammation. In AI, cytokines and reticuloendothelial system cells induce alterations that interfere in different pathways of erythropoiesis leading to anemia.^2^

Pro- and anti-inflammatory cytokines, derived from macrophages or T cells, as well as acute phase proteins, are believed to be involved in AI Fe homeostasis disorders. The demonstration of the importance of IL-1 and TNF-α in Fe homeostasis occurred from experiments with rats, where the administration of these cytokines was associated with hypoferremia, and induction of ferritin synthesis by SRW. It is now known that IL-1 and IL-6 are able to modulate the translation of ferritin acting on the 5'-untranslated portion of the ferritin messenger RNA.^3,4^

IL-6 appears to play a key role in the stimulation of HAMP transcription, although IL-1α and IL-1β also play a role in the transcription of this gene. IL-6 has the ability to bind to the cell membrane through specific receptors, and to activate the signal transducer and transcriptional activator 3 (*Stat3*), the JAK/STAT signaling pathway, which acts on the gene promoter region by stimulating hepcidin transcription. Excessive production of hepcidin occurs in individuals with inflammatory and infectious diseases, particularly Chagas disease, and this excess explains the sequestration of Fe in macrophages, and the inhibition of intestinal absorption of Fe, two hallmarks of AI.^5^

The expression of ferroportin is decreased in SRE cells due to inflammation; it is not only due to the internalization and degradation of ferroportin by the action of hepcidin, but also by a negative regulation of its expression. Alterations in the differentiation and proliferation of erythroid precursors (BFU-E and CFU-E): a blockade is observed due to the inhibitory effect of several cytokines, in particular: interferon-α, β e γ, α tumoral necrosis factor (TNF-α), and interleukin 1 (IL-1). The related mechanism appears to be the induction of apoptosis; however, cytokines also exert a direct toxic effect on progenitor cells by inducing the formation of free radicals.^6^

Chronic chagasic cardiomyopathy (CCC) is the most severe manifestation of Chagas disease (Chd), in which intense and extensive inflammatory and fibrotic action is observed on the myocardium,^1^ causing structural and autonomic alterations that affect approximately 20% to 30% of the infected people.^7,8,9^ In addition, CCC presents, as a fundamental morphological substrate, a chronic, progressive and fibrosing and, consequently, clinical myocarditis, ranging from silent to more severe forms, such as refractory heart failure (HF), complex arrhythmias, ventricular aneurysms, and sudden death.^10^

The impairment of cardiac function, as well as the progression of neurohormonal and inflammatory compensation mechanisms, can either alter iron metabolism by simply reducing its intestinal absorption, or dynamically change its distribution in the reticuloendothelial and hematopoietic system.^7,8,9,6,11,12^

Anemia is known to be the last compensatory stage when there is iron bioavailability impairment for the erythropoietic processes from complex pathophysiological mechanisms. The objective of this study was to check if the iron kinetics markers correlate with the degree of ventricular dysfunction of Chagas cardiomyopathy compared to non-chagasic cardiomyopathy (NCh).

## Methods

### Study population

Forty patients with CCC, 40 chagasic patients with undetermined form (IND), and 40 patients with NCh were consecutively selected according to the inclusion and exclusion criteria. Patients with CCC and with the indeterminate form of Chagas disease showed confirmatory serology for T. cruzi. This study was approved by the Research Ethics Committee of UFMG-COEP with identification number ETIC 359/04.

### Study design and procedure

At the time of inclusion, all patients underwent clinical examination, laboratory examination, 12-lead ECG. Functionalcapacity was assessed by the *New York Heart Association* (NYHA) scales. The severity of cardiac involvement was determined by echocardiographic indices (ejection fraction [EF], and left ventricular end-diastolic volume, LV).

### Hematologic evaluation

Serum iron dosage (FeSe), transferrin saturation index (TSI), total iron-binding capacity (TIBC), and ferritin were sent to the Central Laboratory of Hospital das Clínicas. Their quantifications were done through the following methods:

- serum iron (two-point kinetics)
- transferrin saturation index, ferritin (immunoturbidimetry), were quantified by the calculation (serum iron - total iron-binding capacity).
- total iron-binding fixation capacity (enzymatic kinetics).
- transferrin dosage (immunoturbidimetry).

### Sample size calculation

The work by Jankowska et al. (2012), in which functional classes III and IV HF patients were studied, was the basis for the sampling calculation, and showed alterations in iron metabolism and association with the degree of ventricular systolic dysfunction, and morbidity. Sample size was calculated by the BioStat 5.3 Software, for which the mean and standard deviation [±] of each iron kinetic variable with minimum test power of 0.80 were used, assuming a significance of 0.05% (> 5%) and beta error lower than 20% (test power); we decided to select 40 patients in each IND, CCC and NCh group.

### Statistical analysis

Statistical analysis was performed using SPSS Software version 22.0 (SPSS Inc., Chicago, Illinois, United States). A descriptive analysis of continuous and categorical variables was performed. For the presence or absence of normal distribution of variables, the Shapiro-Wilk test was performed. For the multivariate analysis presented in Table 2, the Cox regression model was used, and the association of variables that were related to death, hazard ratio (HR) and 95% CI was evaluated, assuming a statistical significance of 0.05%.

**Table 2:** Variables of iron kinetics markers independently associated with left ventricular systolic dysfunction below 35% (multivariate Cox Hazard proportional hazard model)

	Univariate model				Multivariate model			
Variables	OR	95% CI	X^2^	p	OR	95% CI	X^2^	p
TSI%	0.89	0.816-0.979	5.86	0.015	1.12	1.02-1.22	6.3	0.012
FeSe	0.97	0.95-0.99	5.34	0.021	1.23	1.00-1.04	5.9	0.014
FERRITIN	1.27	1.044-1.56	5.65	0.017	-	-	-	-
Creatinine	1.01	7.54 ± 13.7	1.36	0.243	-	-	-	-
GFR, mL/min/1.73 m	1.00	0.95-1.05	1.41	0.786	-	-	-	-
Hb	0.99	0.56-1.74	0.001	0.99	-	-	-	-
Anemia	8.97	1.01-7.90	3.90	0.04	1.22	0.126-11.83	0.030	0.862

Univariate and multivariate logistic regression analysis of laboratory parameters. FeSe: serum iron; Hb: hemoglobin; TSI: transferrin saturation index; GFR: glomerular filtration rate.

### Selection criteria applied to the multivariate model

Presence of left ventricular systolic dysfunction (LVEF ≤ 35%). Left ventricular diameter (LVD) < 55mm. FeSe serum iron < 31*µ*g/dL. When the IST transferrin saturation index is less than 20%. TIBC < 250 *µ*g/dL. Ferritin < 200 mg/dL.

## Results

The demographic, clinical, laboratory and echocardiographic characteristics of the groups are presented in Table 1. There was a predominance of male patients, and most of the patients were NYHA I functional class, with mean left ventricular (LV) FE below 45%.

**Table 1:** Demographic, clinical, laboratory and echocardiographic characteristics of the IND, CCC and NCh groups

Characteristics	IND (n = 40)	CCC (n = 40)	NCh (n = 40)	p
Age*	49.68 ± 5.28	50.98 ± 5.88	49.20 ± 10.09	0.929
Height (cm)	1.69 ± 0.065	1.97 ± 0.158	1.94 ± 0.123	0.889
Weight (kg)*	79.100 ± 8.58	73.75 ± 10.15	71.88 ± 11.47	0.621
Male [n(%)]	21/40 (52.2%)	20/40 (50%)	5/40 (12.5%)	0.979
NYHA functional class	-	-	-	-
I	40/40 (100%)	12/40 (30%)	32/40 (80%)	0.410
II	-	15/40 (37.5%)	6/40 (15%)	0.510
III	-	8/40 (2.5%)	0.312	
IV	-	5/40 (12.5%)	1/40(2.5%)	0.112
FeSe (µg/dL)*	125.30 ± 22.79	93.15 ± 36.53	114.77 ± 18.90	0.004
Hb (g/dL)*	14.84 ± 1.56	13.62 ± 1.23	14.02 ± 1.25	0.010
TSI (%)*	30.95 ± 7.06	29.48 ± 6.59	39.70 ± 8.54	0.001
TIBC (µg/dL )*	196.52 ± 56.95	297.30 ± 36.46	275.18 ± 33.48	0.001
Ferritin (ng/mL)**	156.25 (1.7-42.20)	134.5 (1.56-42.36)	112.95 (2.8-42.66)	0.004
LVD (mm)*	46.38 ± 7.34	65.43 ± 7.70	46.38 ± 7.34	0.002
E/e' ratio*	6.6 ± 2.82	14.9 ± 4.58	12.15 ± 12.06	0.001
LVEF**	65.85 ± 5.9	35.92 ± 8.59	34.95 ± 8.12	0.001

NYHA: New York Heart Association functional class; FeSe: serum iron; Hb: hemoglobin; TSI: transferrin saturation index; TIBC: total iron binding capacity; LVD: diameter of the left ventricle in diastole; E/e: diastolic velocity; LVEF: ejection fraction of the left ventricle.

In the univariate analysis, it was found that the variables that were associated with left ventricular systolic dysfunction below 35% were TSI, (OR = 0.89, p = 0.05), iron (OR = 0.97, ferritin (OR = 1.27, p = 0.017), gender (OR = 0.26, p*=*0.05), HF etiology (OR = 2.40, p = 0.011), and anemia (OR = 8.97, p = 0.04). In the multivariate analysis, there was an independent association between low left ventricular dysfunction of 35% and TSI (OR = 1.12, p = 0.012), FeSe (OR = 1.02, p = 0.014), gender (OR = 3.94, p = 0.038), and the etiology of HF (OR = 2.6, p = 0.036); 35 individuals with left ventricular dysfunction (87.5%) were identified, who presented the outcome in the sample.

## Discussion

Iron kinetic markers were found to correlate with the degree of ventricular dysfunction of chagasic cardiomyopathy in relation to NCh; the following observations were obtained as main results: (a) CCC patients, when compared with IND and NCh patients, had lower serum levels of iron, ferritin, TSI and TIBC; (b) patients with CCC have lower serum levels of iron, TSI, TIBC, and ferritin than patients with Chagasic and non chagasic cardiomyopathy; (c) lower serum levels of iron, TSI, TIBC, and ferritin are associated with the level of systolic ventricular dysfunction; (d) low serum levels of iron, TSI, TIBC, and ferritin are associated with the degree of cardiac morbidity.

As demonstrated by the results presented, we observed that patients with CCC, when compared with chagasic patients in the indeterminate form (IND) and those with NCh, present lower serum levels of iron, TIBC, TSI and ferritin. Currently, there are no studies specifically related to CCC and iron metabolism; thus, we will base our pathophysiological hypotheses on studies performed with other causes of cardiomyopathy. Despite the peculiarities of CCC, there seems to be similarity in the genesis of changes in iron metabolism observed in other pathologies and CCC. As possible pathophysiological mechanisms for iron metabolism alterations in HF, some theories have been described, such as chronic inflammation, intestinal loop edema, and hypoperfusion of the gastrointestinal tract (GIT).^5,6,7^ A prospective case control study with 499 patients with chagasic cardiomyopathy reported the persistence of the parasitic element (DNA) through PCR analysis.^7^ There was an association between parasite load and disease severity measured from clinical parameters.^7^

Regarding the analysis of the iron kinetics markers and the echocardiographic variables, we found that the higher the systolic ventricular dysfunction, the lower the serum FeSe, TSI, TIBC, and ferritin levels. This finding is interesting because it reinforces our hypothesis that low levels of iron kinetics markers correlate with the degree of ventricular dysfunction.

In the multivariate analysis, we found low levels of FeSe (p = 0.014) and TSI (p = 0.012), as well as statistically significant results for gender (p = 0.038) as independent markers for left ventricular systolic dysfunction. The fall of ten units of iron is associated with a 23% higher chance of occurrence of systolic ventricular dysfunction. The fall of ten units of TSI is associated with a 12% higher chance of occurrence of systolic ventricular dysfunction.

## Conclusion

In the population studied, analyzes of iron metabolism in patients with CCC showed that there is an association with the degree of myocardial impairment, and the lower the iron serum levels, the total iron binding capacity, the transferrin saturation index and ferritin, the greater the degree of ventricular dysfunction. It is concluded that in chagasic cardiopathy there is a change in the iron metabolism, and it is more pronounced than in non-chagasic cardiopathies, thus evidencing its infectious nature.
